# Metastatic renal cell carcinoma presenting with melena

**DOI:** 10.1002/ccr3.1492

**Published:** 2018-03-24

**Authors:** Ashwini Sadhale, Abimbola Adike, Dora Lam‐Himlin

**Affiliations:** ^1^ Department of Gastroenterology and Hepatology Mayo Clinic Scottsdale Arizona; ^2^ Department of Pathology Mayo Clinic Scottsdale Arizona

**Keywords:** Melena, metastasis, pancreas, renal cell cancer, small bowel

## Abstract

Renal cell carcinoma is a highly malignant neoplasm. Metastasis to the pancreas and gastrointestinal tract is rare. In this case report, we show images of metastatic renal cell carcinoma to the upper gastrointestinal tract in a patient who presented with melena.

## Case Report

**Figure 1:**
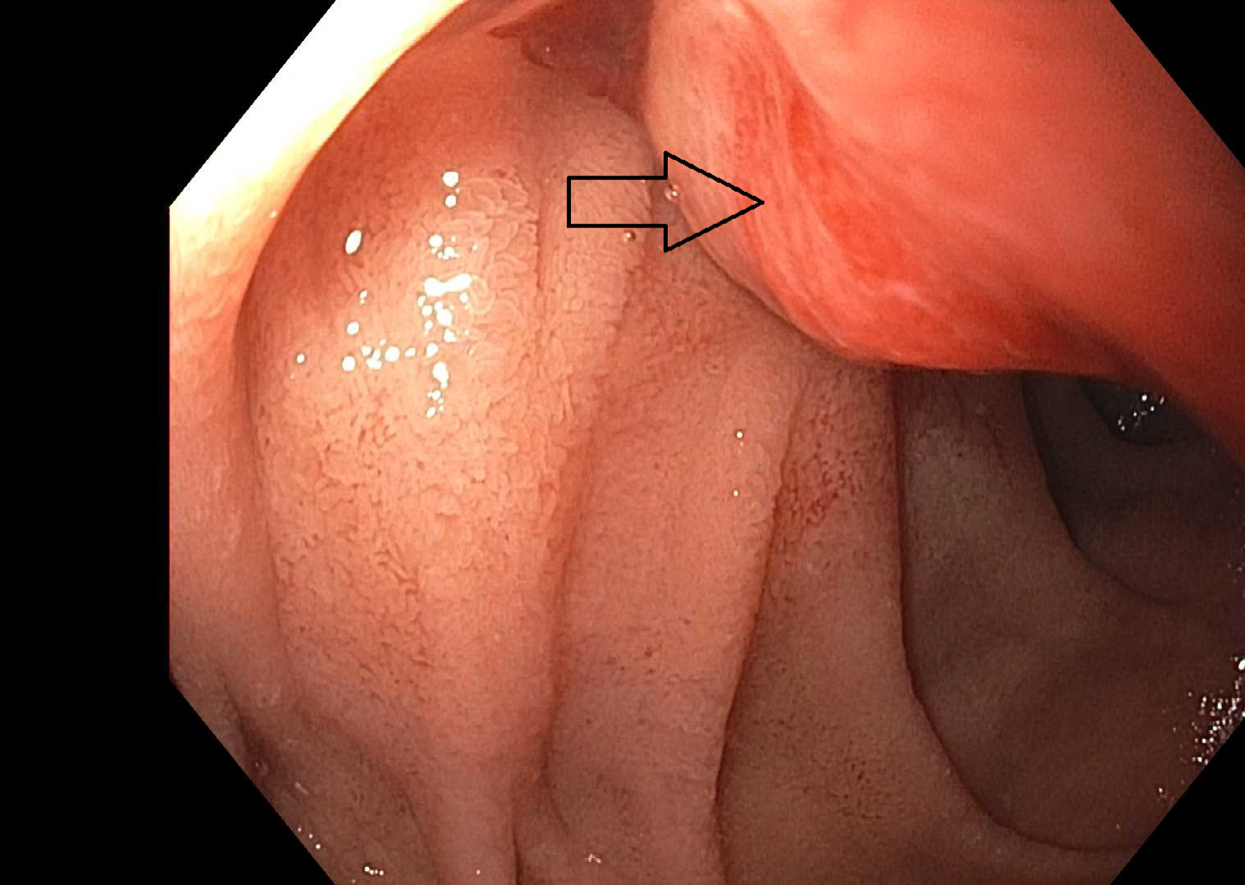
Esophagogastroduodenoscopy (EGD) showing a large friable, sessile, and nonbleeding mass in the duodenal sweep.

**Figure 2:**
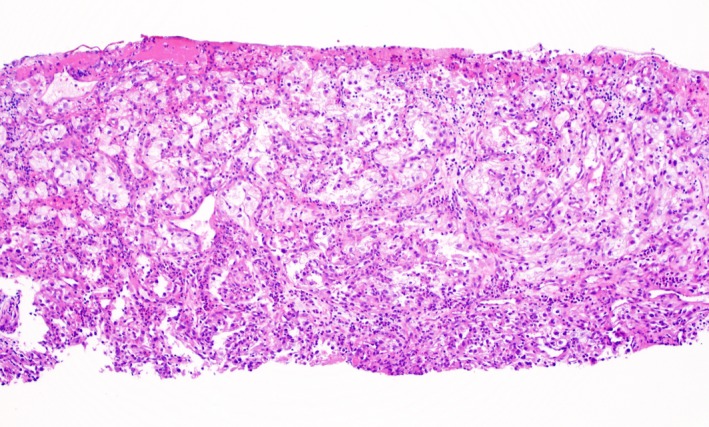
Fragments of an ulcer bed without normal duodenal epithelium (hematoxylin and eosin; original magnification 100x).

**Figure 3:**
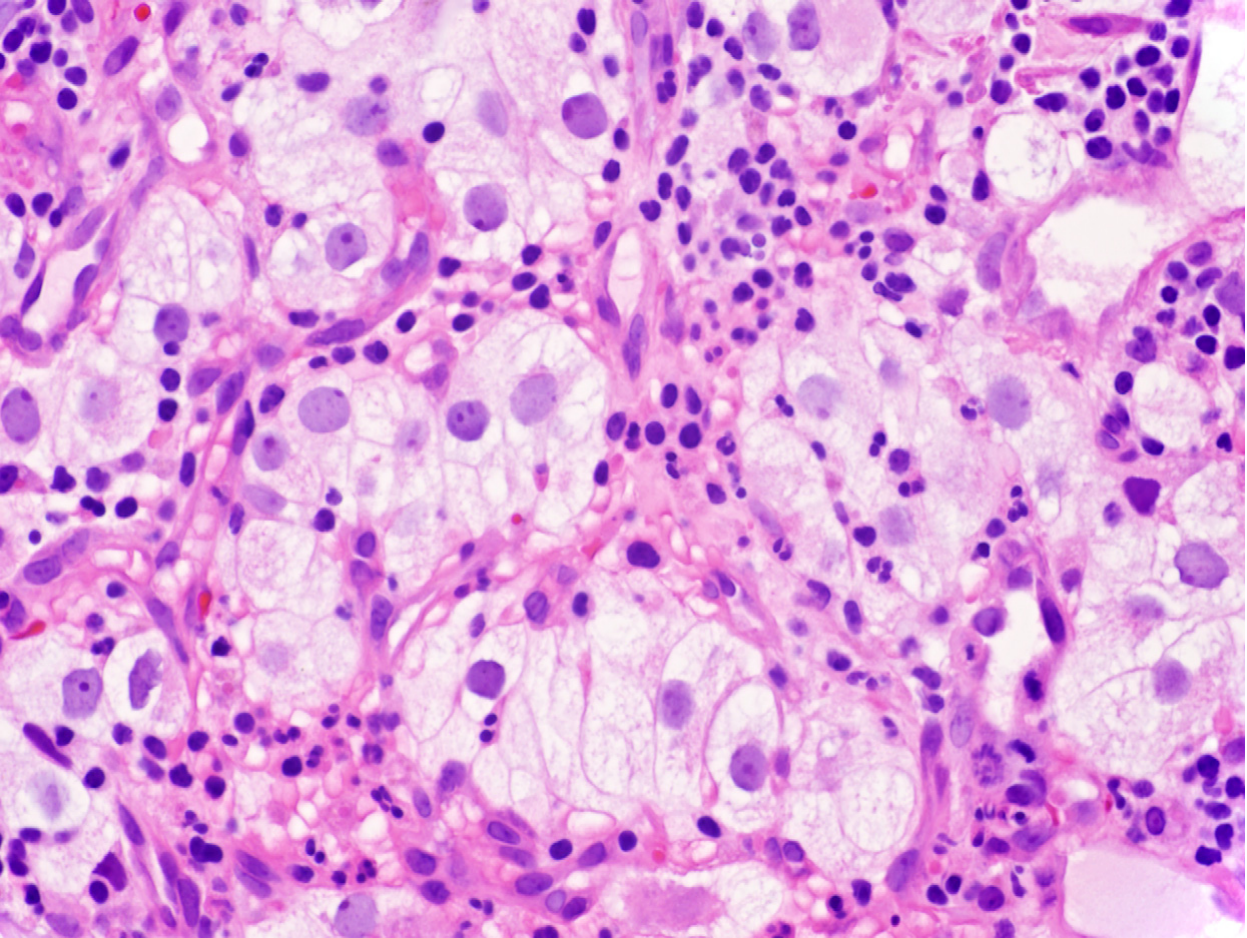
Abundant nests of large clear cells with nested architecture and prominent vasculature (hematoxylin and eosin; original magnification 600x).

**Figure 4:**
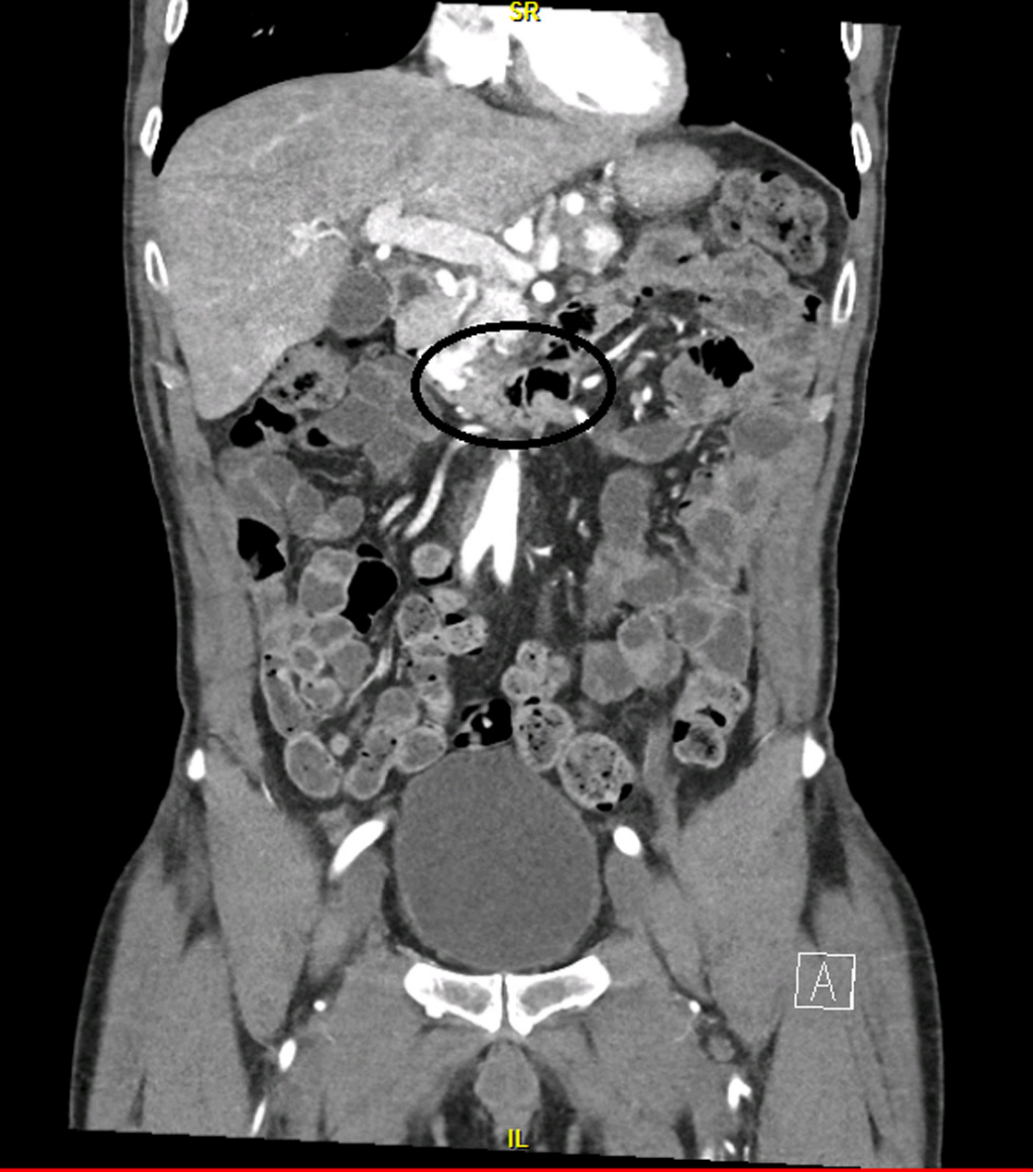
CT of the abdomen showing new intraluminal metastasis invading into the duodenum arising from the largest pancreatic head metastasis measuring 2.5 cm.

A 59‐year‐old gentleman with a history renal cell carcinoma who had undergone right nephrectomy subsequently developed metastasis 13 years later to the lymph nodes, pancreas, thyroid, lungs, and adrenal glands. He had not been treated with radiation or chemotherapy. He presented to the emergency room with 4 days of recurrent melena and lightheadedness. Hemoglobin was 6.2 g/dL from a prior of 12.3 g/dL 2 months earlier. Blood urea nitrogen (BUN) was 38 mg/dl (normal 8–24 mg/dL) and creatinine 1.1 mg/dL (normal 0/8–1.3 mg/dL). On esophagogastroduodenoscopy (EGD), we found that he had a large‐sized friable and sessile mass with no active bleeding at the duodenal sweep (Fig. 1). Histopathology revealed fragments of an ulcer bed without normal duodenal epithelium (hematoxylin and eosin; original magnification 100x, Fig. 2) and abundant nests of large clear cells with nested architecture and prominent vasculature consistent with metastatic renal cell carcinoma (hematoxylin and eosin; original magnification 600x, Fig. 3). Following EGD, a contrast CT of the abdomen showed new intraluminal metastasis invading into the duodenum arising from the largest pancreatic head metastasis measuring 2.5 cm (Fig. 4) without active bleeding. The patient was treated with palliative radiation therapy.

## Discussion

Renal cell carcinoma (RCC) is the most common malignant neoplasm of the kidney 1, 2. This malignancy is known to metastasize several years after the primary tumor has been treated 2. The rate of metastasis prior to treatment of the primary is about 24–28% but may increase to as much as 51% postnephrectomy 2. Common sites of metastasis include the lung and bone while lymph nodes, the brain, liver, and the contralateral kidney are less common sites 2. Metastasis to the pancreas and gastrointestinal tract is rare 2. The least likely site to be affected by metastatic RCC among the GI organs is the duodenum. Metastasis to the small bowel often presents with GI bleeding 3, but may rarely present with obstruction or intussusception 1, 4. In our case, it is likely that the duodenal mass seen on endoscopy and imaging (Figs 1 and 4) arose as a result of direct invasion from a pancreas met.

Renal cell carcinoma can be unpredictable. Metastasis to the small bowel is a potential source of bleeding in patients presenting with GI bleeding who have a history of renal cell carcinoma.

## Authorship

AS and AA: authored and edited the manuscript. AA: finalized the manuscript. DL‐H: provided the histopathological data and overall guidance.

## Conflict of Interest

None declared.
